# Effects of muscle quantity, muscle quality, and phase angle on whole-body reaction time in 5164 adults aged 20–91 years

**DOI:** 10.1007/s00421-024-05591-3

**Published:** 2024-09-08

**Authors:** Wataru Fukuda, Takeshi Yoshihisa, Yosuke Yamada

**Affiliations:** 1 Yokohama Sports Medical Center, Yokohama, Japan; 2 Institute for Active Health, Kyoto University of Advanced Science, Kyoto, Japan; 3 Department of Physical Activity Research, National Institute of Health and Nutrition, National Institutes of Biomedical Innovation, Health and Nutrition, 3-17 Senrioka-shinmachi, Settu-city, Osaka, 566-0002 Japan

**Keywords:** Whole-body reaction time, Muscle quantity, Muscle quality, Bioelectrical impedance analysis

## Abstract

**Purpose:**

Muscle quantity, defined as appendicular lean mass (ALM); muscle quality, defined as the ratio of muscle strength to ALM; and bioelectrical impedance analysis (BIA)-derived phase angle (PhA) are determinants of physical performance. We examined whether muscle quality indices were significant predictors of the whole-body reaction time (WBRT) in healthy female and male adults aged 20–91 years.

**Methods:**

Data from 5164 adults (2869 women and 2295 men; mean age ± standard deviation, 60.9 ± 15.6 years) were analyzed. Height and weight were measured, and body mass index was calculated. ALM was estimated using a previously validated 8-electrode multi-frequency BIA. PhA was measured at 50 kHz using a BIA device. Knee extension strength (KES), leg extension power (LEP), and flexibility were examined. The ALM to weight (ALM/weight), KES to ALM (KES/ALM), and LEP to KES (LEP/KES) ratios were calculated. In the WBRT test, participants were asked to stand on a force plate and jump upright as quickly as possible in response to a light stimulus. The WBRT was divided into the response initiation and motion execution phases.

**Results:**

ALM/weight, KES/ALM, LEP/KES, PhA, and flexibility were significant independent predictors of WBRT and the time of the motion execution phase (*p* < 0.001). However, PhA was not a significant predictor of the time of response initiation phase.

**Conclusion:**

Muscle quantity (ALM/weight), muscle quality (KES/ALM and LEP/KES), PhA, and flexibility are determinants of WBRT test performance, particularly in the motion execution phase.

## Introduction

The whole-body reaction time (WBRT) test is a physical fitness test that has been conducted since the 1950s or 60 s worldwide (Cureton 1951; Karpovich et al. 1960; Ikai et al. 1961), and has been popularly implemented throughout Japan until the present day (Cao et al. 2007; Miyatake et al. 2007, 2012; Sasai et al. 2010; Kai et al. 2012; Makizako et al. 2013; Miyaguchi et al. 2013; Kozakai et al. 2020; Kawakami et al. 2021; Momma et al. 2021). In the WBRT test, participants are asked to stand on a force plate and jump upright as quickly as possible in response to a light stimulus that was 2 m away from their body; the time between the light stimulus and disappearance of foot pressure from the force plate is measured. The WBRT can be divided to two phases: one is a “response initiation phase”, defined as the time required to initiate a movement following the visual signal; and other is a “motion execution phase”, which is defined as the time from the initiation of the movement to the moment when both feet completely leave the ground (Kai et al. 2012). The WBRT assesses the motor abilities of the human body. With the WBRT test, it is possible to measure a velocity index to optimize exercise speed, and, simultaneously, the neurotransmission process and muscle contraction process during exercise can be analyzed separately.

WBRT tests have been conducted in large epidemiologic studies that examined the relationship between physical performance and health outcomes in Japan (Miyatake et al. 2007, 2012; Kai et al. 2012; Makizako et al. 2013; Miyaguchi et al. 2013; Kozakai et al. 2020; Kawakami et al. 2021; Momma et al. 2021). These studies found that the WBRT test is a predictor of future health outcomes. In addition, some studies found that exercise interventions enhance WBRT performance (Cao et al. 2009; Sasai et al. 2010). However, the physiologic determinants of WBRT such as muscle quality, muscle quantity, and flexibility remain unclear.

Muscle quality is considered a determinant of physical performance, independent of muscle quantity (e.g., appendicular lean mass [ALM]) (Akamatsu et al. 2022; Yamada 2018). In 2018, the European Working Group on Sarcopenia in Older People defined muscle quality as (1) the infiltration of fat into the muscle or the attenuation of the muscle by imaging methods such as magnetic resonance imaging or computed tomography; (2) the ratio of muscle strength to ALM; or (3) bioelectrical impedance analysis (BIA)-derived phase angle (PhA) measurement (Cruz-Jentoft et al. 2019). BIA can noninvasively obtain tissue electrical parameters (resistance and reactance) by applying a weak current to the body (Yamada et al. 2009, 2021). Resistance is strongly related to the water content and ion concentration inside and outside the cell membrane, whereas reactance is strongly related to the capacitance of the cell membrane (Barbosa-Silva et al. 2005). The PhA is an index calculated using these parameters. Previous studies have reported that PhA is associated with maximal knee extension (Wada et al. 2020; Hirata et al. 2022) and whole-leg muscle power (Yamada et al. 2017a). Hirata et al. (2022) reported a negative correlation between PhA and the time to reach peak torque during maximal muscle exertion by electrical stimulation. This suggests that the PhA may influence the velocity index during force or motion exertion. However, the relationship between PhA and neurotransmission processes is unclear because muscle contraction induced by electrical stimulation does not include neurotransmission processes in the central nervous system. In addition, because there is a trade-off between force and velocity in force production, the results of force- and velocity-optimized movements may differ (Morin and Samozino 2016; Jiménez-Reyes et al. 2019).

We hypothesized that a greater body PhA would be associated with shorter WBRT, independent of ALM, the ratio of muscle strength to ALM, the ratio of muscle power to muscle strength, and flexibility. Furthermore, it is expected to be more related to the “motion execution phase” than the “response initiation phase”. This study aimed to clarify the relationship between PhA and exercise with optimized movement speed and its relationship with neurotransmission and muscle contraction during exercise through the measurement of WBRT.

## Materials and methods

### Participants

This study was conducted using data from the Sports Program Service study conducted at the Yokohama Sports Medical Center (Ohta et al. 2022). Of these, 5164 adult patients (2295 men and 2869 women) aged 20–91 years from January 2013 to March 2019 were included in the analysis. If one or more measurements were missing for any reason, they were excluded from the analysis. Participants were defined as those who were able to walk, bicycle, or drive to and from home, the nearest train station, or bus stop; did not require the assistance of a caregiver; and had no problems with activities of daily living. Participants provided written informed consent for the use of their data. This study was approved by the Ethics Committee of Yokohama Sports Medical Center (k-2022–002).

### Body composition and PhA

Height and weight were measured using an automatic height and weight scale (WB-510; TANITA, Tokyo, Japan), and body composition was assessed using an 8-point tactile electrode multifrequency body composition scale (MC-190; TANITA). Participants were instructed to stand barefoot on toe-and-heel electrodes and hold the handgrips with the arms hanging down a few centimeters from the hip. The electrical current was ≤ 90 μA, and the minimum weight graduation was 0.1 kg. The details of the BIA device have been described previously (Yamada et al. 2017b). ALM was obtained from a previously validated equation against dual-energy X-ray absorptiometry (Yamada et al. 2017b). PhA was calculated as arctangent (Xc/R) × 180°/π (Gonzalez et al. 2016). Anthropometric and BIA measurements were completed between 9 and 10 am.

#### WBRT

**Fig. 1:**
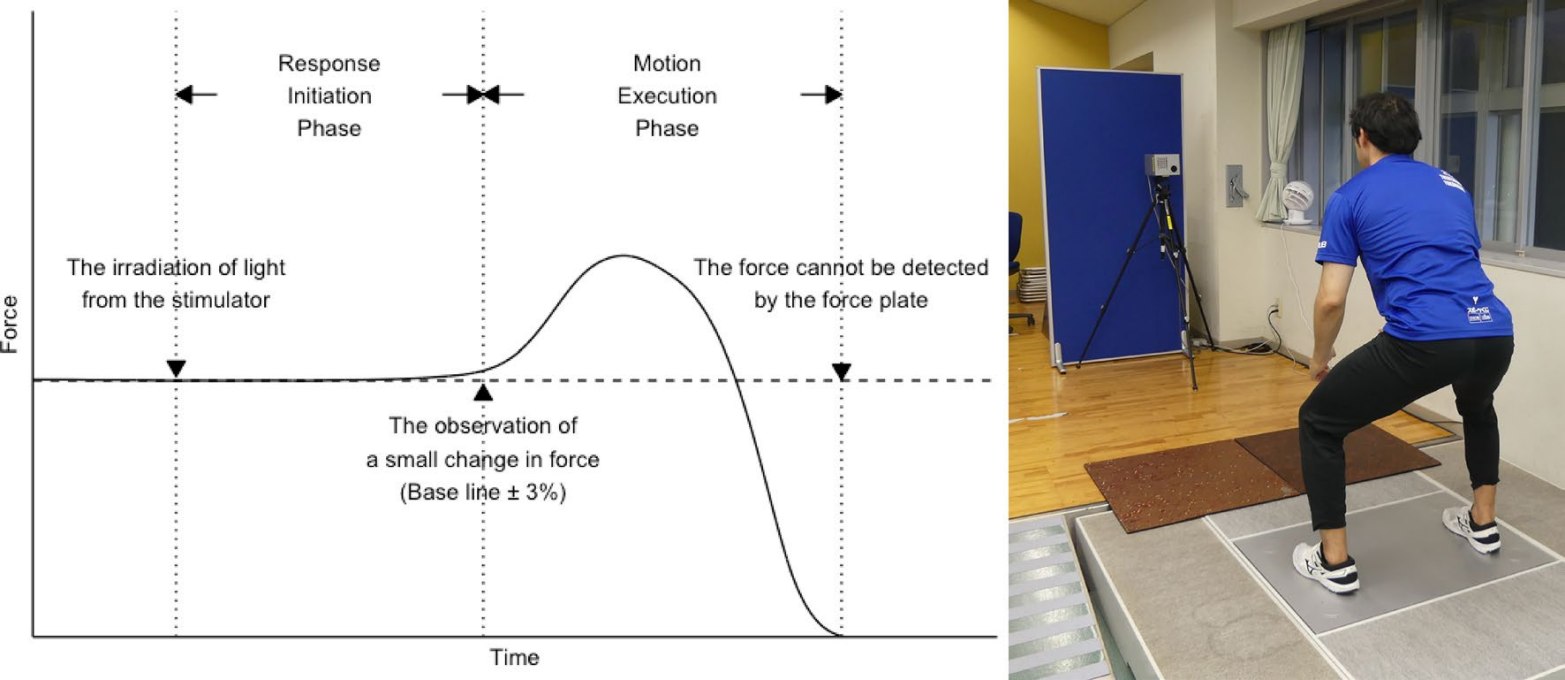
Conceptual diagram and a photograph during the experiment of the whole-body reaction time (WBRT) test

WBRT was measured by standing in a relaxed posture on the force plate and jumping vertically in response to light emitted from the stimulator (Cao et al. 2007; Miyatake et al. 2007, 2012; Sasai et al. 2010; Kai et al. 2012; Makizako et al. 2013; Miyaguchi et al. 2013; Kozakai et al. 2020; Kawakami et al. 2021; Momma et al. 2021). The “response initiation phase” was defined as the time between the irradiation of light from the stimulator and the observation of a small change in force, and the “motion execution phase” was defined as the time between the detection of a small force and the time when the force was no longer detectable on the force plate (Fig. 1). Response initiation was defined as the time during which the numerical value deviated from the baseline by more than ± 3% for 5 ms consecutively from the detected load on the force plate. If the values continued above or below the threshold within a fixed period, the values were above the threshold. The distance between the stimulator and the center point of the force plate was set at 3 m.

### Knee extension strength (KES)

KES was measured using an isokinetic dynamometer (Cybex Humac Norm 770; Computer Sports Medicine Inc., Stoughton, MA, USA) with a knee joint angular velocity of 60°/s during several trials of two to three consecutive knee joint extension and flexion movements. Peak torque during knee joint extension was recorded for each trial, and the highest value in the measured trials was expressed as a percentage of body weight (Nm/kg). The ratio of KES to ALM (KES/ALM) was calculated as the muscle quality index. Details of the KES measurements are described elsewhere (Ohta et al. 2022).

### Leg extension power (LEP)

Lower limb muscle power (Yamada et al. 2023) was evaluated as LEP using a constant-velocity leg extension muscle tester (LEG POWER; Takei Scientific Instruments Co., Ltd., Niigata, Japan) with the footplate set to move at 0.8 m/sec. The highest value among the measurement trials was taken as the representative value and expressed as a percentage of body weight (W/kg). The ratio of LEP to KES (LEP/KES) was calculated as one of muscle quality index. Details of the LEP measurements have been described elsewhere (Yamada et al. 2023).

### Flexibility

Flexibility was assessed by bending forward in a sitting position by using a measuring instrument (SIT-AND-REACH; Takei Scientific Instruments Co.) (Kimura et al. 2012). A mat was placed next to the measuring instrument at a height of approximately 5 cm above the ground. The patients sat with their legs stretched out on the mat, the soles of their feet firmly placed on the measuring instrument, and bent forward so that both knees would not bend. Measurements were taken in 0.5 cm increments with the plantar surface as the reference, with the far side being positive and the front side being negative. All subjects were measured two to three times, with the best record being representative.

### Statistical analyses

All analyses were performed using SPSS software (version 28.0 for Windows; IBM Corp. Armonk, NY, USA). The results are presented as means and standard deviations. T tests were used for comparisons between women and men. Partial correlation coefficients were obtained between WBRT, response initiation phase or motion execution phase, and the independent variables with age, sex, and body mass index (BMI) as control variables. Multiple linear regression analyses were performed with WBRT, response initiation phase or motion execution phase as the dependent variables. A variance inflation factor (VIF) of > 5 indicated that the regression coefficients are estimated in the presence of multicollinearity. The statistical significance value was set at *p* < 0.001 in the current study because the large sample size (*n* = 5164) decreases the risk of Type II error but increased the risk of Type I error.

## Results

Table 1 shows the physical characteristics of women and men in the participants. The time of the response initiation phase was not significantly different between women and men; however, the other variables were significantly different between women and men (*p* < 0.001). Percent fat and trunk flexibility were significantly greater and WBRT and motion execution phase times were significantly longer in women than in men (*p* < 0.001). In contrast, PhA, KES, LEP, ALM, ALM/weight, KES/ALM, and LEP/KES were significantly higher in men than in women (*p* < 0.001).

**Table 1:** Physical characteristics in the 5164 participants aged 20–91 y (mean ± SD)

	Women (*n* = 2869)	Men (*n* = 2295)	*p* value
Age (y)	62.1 ± 14.4	59.4 ± 16.8	< 0.001
Height (cm)	155.0 ± 6.0	168.6 ± 6.2	< 0.001
Weight (kg)	50.8 ± 7.6	64.8 ± 10.2	< 0.001
BMI	21.2 ± 2.8	22.7 ± 3.0	< 0.001
%fat (%)	26.7 ± 6.7	18.1 ± 5.9	< 0.001
WBRT (msec)	372 ± 37	356 ± 35	< 0.001
Response-initiation phase (msec)	187 ± 24	185 ± 23	n.s
Motion-execution phase (msec)	186 ± 31	171 ± 27	< 0.001
PhA (deg)	5.10 ± 0.57	5.88 ± 0.7	< 0.001
KES (Nm/kg)	86 ± 20	147 ± 40	< 0.001
LEP (W/kg)	401 ± 105	716 ± 214	< 0.001
Flexibility (cm)	11.6 ± 8.4	2.6 ± 11.0	< 0.001
ALM (kg)	15.8 ± 2.0	23.1 ± 3.6	< 0.001
SMI (ALM/Height^2^) (kg/m^2^)	10.2 ± 1.0	13.7 ± 1.7	< 0.001
ALM/weight (%)	31.4 ± 3.6	36.0 ± 3.9	< 0.001
KES/ALM	5.43 ± 0.98	6.33 ± 1.25	< 0.001
LEP/KES	4.69 ± 0.86	4.90 ± 0.90	< 0.001

*BMI* body mass index, *%fat* percent body fat, *WBRT* whole-body reaction time, *PhA* phase angle, *KES* knee extension strength, *LEP* leg extension power, *ALM* appendicular lean mass

Table 2 shows the partial correlation coefficients between WBRT and the independent variables. PhA was significantly and negatively correlated with WBRT and time of the motion execution phase (*p* < 0.001) but not with time of the response initiation phase. ALM was not significantly correlated with either the response initiation phase or the motion execution phase. ALM/Weight was not significantly correlated with the response initiation phase. Other variables were significantly correlated with the WBRT, response initiation phase, and motion execution phase (*p* < 0.001). The WBRT test performance was more highly correlated with ALM/weight than with ALM or SMI with respect to muscle quantity indices. ALM, SMI, and ALM/weight were highly correlated (*r* > 0.8, *p* < 0.001). Therefore, we used the ALM/weight variable in the subsequent multiple regression analyses to avoid multicollinearity.

**Table 2:** Partial correlation coefficients between reaction time and independent variables

	WBRT (msec)	Response-initiation phase (msec)	Motion-execution execution phase (msec)
PhA (deg)	−0.159*	−0.018	−0.176*
KES (Nm/kg)	−0.235*	−0.162*	−0.149*
LEP (W/kg)	−0.298*	−0.180*	−0.210*
Flexibility (cm)	−0.159*	−0.092*	−0.116*
ALM (kg)	−0.047*	−0.035	−0.028
SMI (ALM/Height^2^)	−0.082*	−0.036	−0.069*
ALM/Weight (%)	−0.126*	−0.030	−0.127*
KES/ALM	−0.249*	−0.176*	−0.154*
LEP/KES	−0.128*	−0.050*	−0.113*

Control variables: age, sex and BMI

*WBRT* whole-body reaction time, *PhA* phase angle, *KES* knee extension strength, *LEP* leg extension power, *ALM* appendicular lean mass

*p < 0.001

Table 3 shows the results of multiple linear regression analyses for the WBRT, response initiation phase, and motion execution phase. The VIFs of all variables were < 5. ALM/weight, KES/ALM, and LEP/KES were significant predictors of WBRT (*p* < 0.001). PhA was a significant predictor of WBRT and time of the motion execution phase, independent of age, sex, BMI, ALM/weight, KES/ALM, LEP/KES, and flexibility (*p* < 0.001). PhA was not a significant predictor of the time of the response initiation phase.

**Table 3:** Multiple linear regression analyses for WBRT, response initiation phase, and motion execution phase

		WBRT (msec)			Response-initiation phase (msec)			Motion-execution execution phase (msec)		
		Un-standardized	Standardized	*p* value	Un-standardized	Standardized	*p* value	Un-standardized	Standardized	*p* value
	VIF	B	β		B	β		B	β	
Constant		519.822		< 0.001	249.542		< 0.001	270.493		< 0.001
Age (y)	1.783	0.129	0.054	< 0.001	0.020	0.013	n.s	0.109	0.056	< 0.001
Sex (F = 0, M = 1)	3.278	−1.881	−0.025	n.s	−4.623	−0.098	< 0.001	2.722	0.045	n.s
BMI	3.174	0.920	0.075	< 0.001	−0.177	−0.023	n.s	1.093	0.109	< 0.001
ALM/Weight (%)	4.524	−1.292	−0.153	< 0.001	−0.528	−0.098	< 0.001	−0.767	−0.111	< 0.001
KES/ALM	1.953	−11.784	−0.380	< 0.001	−5.556	−0.281	< 0.001	−6.238	−0.245	< 0.001
LEP/KES	1.199	−9.432	−0.225	< 0.001	−3.456	−0.129	< 0.001	−5.989	−0.175	< 0.001
Flexibility (cm)	1.320	−0.276	−0.080	< 0.001	−0.122	−0.055	< 0.001	−0.154	−0.054	< 0.001
PhA (deg)	2.665	−4.118	−0.082	< 0.001	1.687	0.053	n.s	−5.787	−0.141	< 0.001

Adj. R2 = 0.289, 0.076, and 0.223, and for WBRT, response initiation phase, and motion execution phase, respectively

*WBRT* whole-body reaction time, *ALM* appendicular lean mass, *KES* knee extension strength, *LEP* leg extension power, *PhA* phase angle

## Discussion

We found that ALM/weight, KES/ALM, LEP/KES, and PhA were significant predictors of WBRT performance, particularly during the motion execution phase, independent of age, sex, BMI, and flexibility. ALM/weight, KES/ALM, and LEP/KES were statistically significant predictors of the time of the response initiation phase; however, the adjusted coefficient of determination in the multiple linear regression analyses was too small (adjusted R^2^ = 0.076). Muscle quantity, quality, and PhA were the determinant factors of the motion execution phase of the WBRT test rather than the response initiation phase.

Multiple regression analyses indicated that ALM/weight (an index of the ratio of muscle mass to body mass), KES/ALM (an index of the ratio of muscle strength to muscle mass), LEP/KES (an index of the ratio of muscle power to muscle strength), and PhA (an index of muscle cell quality or muscle tissue composition) were significant independent predictors of WBRT performance. The current results suggest that muscle quantity and quality are possible determinants of the kinetic speed of the motion execution phase in the WBRT. Muscle mass is generally considered to be the primary factor affecting muscle strength and/or power. However, some researchers (Yamada 2018) noted a discrepancy in the rate of decrease in muscle mass, 0.5%–1% per year, versus 1%–4% per year for muscle strength and power. This suggests that muscle mass, strength, and power are not interchangeable. The strength-to-mass and power-to-strength ratios of muscles are important determinants of the kinetic speed of the vertical jump motion in the WBRT. In particular, KES/ALM showed the largest standardized beta in multiple linear regression analysis.

Interestingly, PhA was a significant and independent predictor of WBRT test performance, independent of muscle quality indices, defined as the strength-to-mass and power-to-strength ratios. A previous study demonstrated that PhA is correlated with a muscle quality index defined by the strength-to-mass ratio (Akamatsu et al. 2022). Similarly, PhA was significantly correlated with the KES/ALM (*r* = 0.448, *p* < 0.001; Pearson’s correlation coefficient) in the present study. However, almost no correlation was observed between PhA and KES/ALM in the partial correlation analysis, with sex, age, and BMI as control variables (*r* = 0.094). Previous studies have indicated that PhA is a determinant of various physical performances independent of muscle strength (Yamada et al. 2017a, 2021; Yoshida et al. 2018; Hirata et al. 2022). However, the physiologic mechanisms underlying the relationship between PhA and muscle function remain unclear. A previous study demonstrated that leg PhA can be an indicator of voluntary and evoked muscle contractile properties but not of the neuromuscular activity of the plantar flexors (Hirata et al. 2023).

One limitation of the current study is that the study design was cross-sectional and not longitudinal or interventional; thus, we could not prove causality. Another limitation was that the adjusted coefficient of determination in the multiple linear regression analyses for WBRT was relatively moderate or low (R^2^ = 0.289). This is because the reaction time tests have large intra- and inter-individual variabilities between trials. Further studies are required to examine the physiologic and psychologic determinants of WBRT performance. However, to the best of our knowledge, this is the first study to examine the physiologic factors that determine WBRT test performance using a large sample size.

In conclusion, muscle quantity, muscle quality, PhA, and flexibility are significant and independent determinants of WBRT test performance, particularly during the motion execution phase, in healthy adults aged 20–91 years. Previous epidemiologic studies have shown that the WBRT test predicts future health outcomes. In addition, some studies have shown that exercise interventions enhance the WBRT test performance. In the present study, we explored the physiologic determinants of WBRT. Further studies are needed to fully elucidate the association between WBRT and health outcomes.

## Abbreviations

ALM: Appendicular lean mass
BIA: Bioelectrical impedance analysis
BMI: Body mass index
KES: Knee extension strength
LEP: Leg extension power
PhA: Phase angle
VIF: Variance inflation factor
WBRT: Whole-body reaction time
